# Evaluation of Potential Probiotics *Bacillus subtilis* WB60, *Pediococcus pentosaceus*, and *Lactococcus lactis* on Growth Performance, Immune Response, Gut Histology and Immune-Related Genes in Whiteleg Shrimp, *Litopenaeus vannamei*

**DOI:** 10.3390/microorganisms8020281

**Published:** 2020-02-19

**Authors:** Seonghun Won, Ali Hamidoghli, Wonsuk Choi, Jinho Bae, Won Je Jang, Seunghan Lee, Sungchul C. Bai

**Affiliations:** 1 Department of Marine Bio-materials and Aquaculture/Feeds & Foods Nutrition Research Center (FFNRC), Pukyong National University, Busan 608–737, Korea; ks0sk@naver.com (S.W.); alihamid@pukyong.ac.kr (A.H.); thm622@naver.com (W.C.); bjh2921@naver.com (J.B.); shlee5863@naver.com (S.L.); 2 Department of Biotechnology, Pukyong National University, Busan 608–737, Korea; jangwj9914@naver.com

**Keywords:** Probiotic, feed additive, *Bacillus*, *Lactococcus*, *Pediococcus*, whiteleg shrimp

## Abstract

An eight-week feeding trial was conducted to evaluate the effects of different dietary probiotic supplements in juvenile whiteleg shrimp, *Litopenaeus vannamei*. A basal control diet without probiotics (CON), and five other diets by supplementing *Bacillus subtilis* at 10^7^ CFU/g diet (BS_7_), *B. subtilis* (BS_8_), *Pediococcus pentosaceus* (PP_8_), and *Lactococcus lactis* (LL_8_) at 10^8^ CFU/g diet, and oxytetracycline (OTC) at 4 g/kg diet were used. Whiteleg shrimp with initial body weights of 1.41 ± 0.05 g (mean ± SD) were fed with these diets. Growth of shrimp fed BS_8_ and LL_8_ diets was significantly higher than those of shrimp fed the CON diet (*p* < 0.05). Superoxide dismutase activity in shrimp fed PP_8_ and LL_8_ diets was significantly higher than that of shrimp fed the CON diet (*p* < 0.05). Lysozyme activity of shrimp fed probiotics and OTC diets significantly improved compared to those on the CON diet (*p* < 0.05). The intestinal histology showed healthier guts for shrimp fed the probiotic diets (*p* < 0.05). Immune-related gene expression in shrimp fed BS_8_, PP_8_ and LL_8_ diets was recorded as significantly higher than that of shrimp fed CON and OTC diets (*p* < 0.05). Also, results of the challenge test for 7 days and the digestive enzyme activity of shrimp fed BS_8_, PP_8_, and LL_8_ were significantly improved compared to those on the CON diet (*p* < 0.05). Therefore, these results indicated that *L. lactis* at 10^8^ CFU/g could be an ideal probiotic for whiteleg shrimp, and also *B. subtilis* WB60 and *P. pentosaceus* at 10^8^ CFU/g could improve the growth, immunity, histology, gene expression, digestive enzyme activity, and disease resistance, while replacing antibiotics.

## 1. Introduction

The whiteleg shrimp, *Litopenaeus vannamei*, is a commercially important shrimp species which accounts for 80% of global shrimp production. The global production of whiteleg shrimp has increased rapidly and reached 445 million metric tons in 2017, with an estimated total value of 26.7 billion US dollars [1]. With the high market demand for whiteleg shrimp, it has been cultured intensively, which has led to serious problems such as infectious disease outbreaks by parasites. In particular, *Vibrio* bacterium is the most frequent pathogen in shrimp farming, which has a serious impact on survival, immune responses, and production losses [2]. The emergence of infectious pathogen diseases in shrimp farming causes the abuse or misuse of antibiotic agents [3,4]. Antibiotics are used as chemotherapeutic agents for shrimp because they can influence an extensive range of gram-negative/positive bacteria. However, the excessive use of antibiotics can result in the occurrence of bacterial resistance [5]. Moreover, the use of antibiotics in shrimp aquaculture has drawn attention from a public health point of view because of the potential risks and issues for human consumers [6]. Consequently, studies on possible replacements for antibiotics in shrimp aquaculture are required.

The benefits of dietary probiotics on growth, immunity, disease resistance, and digestive enzyme activity in shrimp aquaculture have been reported by several authors [7,8]. In addition, according to previous studies [9,10], probiotics are safe approaches used as antibiotic replacers in shrimp aquaculture practices. In fact, probiotics play an important role in preventing and maintaining the microbial balance between necessary and excessive defense mechanisms in terms of innate immune responses [11]. Many bacterial species such as *Bacillus subtilis* [12,13], *Pediococcus pentosaceus* [14,15], and *Lactococcus lactis* [16,17] have been demonstrated as growth, immune response, and disease resistance promoters in shrimp cultures. Some of these bacteria naturally occur in gastrointestinal tract of fish and are able to produce substances with antibacterial activity [18]. As a result, they can increase growth, feed efficiency and immune responses of fish. However, previous studies only evaluated the effects of each of these probiotics and have not compared probiotics with antibiotics.

Therefore, the present study aimed at comparing dietary *B. subtilis* WB60 that was previously extracted and isolated form Japanese eel *Anguilla japonica* intestine, with *P. pentosaceus,* and *L. lactis* that had been obtained from the whiteleg shrimp digestive tract. Also, comparisons were made with a commonly used antibiotic (oxytetracycline) in shrimp aquaculture. 

## 2. Materials and Methods

The study was conducted under the guidelines of the Animal Ethics Committee Regulations, No.18–0145 issued by the Pukyong National University, Busan, Rep. Korea.

### 2.1. Probiotic Selection

The selected probiotic strains evaluated in this study were isolated from the intestine of Japanese eel [18] and shrimp [15,17]. Among the probiotics, *B. subtilis* was isolated from the intestine of juvenile Japanese eel and was identified as *B. subtilis* WB60 according to [17]. This probiotic was incubated at 30 °C for 72 h in LB broth (Sigma-Aldrich, St. Louis, MO, USA) and measured at 600 nm optical density (OD_600_) using spectrophotometer. *P. pentosaceus* and *L. lactis* were isolated from the intestine of juvenile whiteleg shrimp [15,17]. The strain *P. pentosaceus* was cultured and incubated at 25 °C for 48 h in TSA. While *L. lactis* was grown in MRS (De Man Rogosa & Sharp broth) medium and incubated at 30 °C for 48 h. Three probiotics were washed in sterile saline and the concentration of the final suspension was calculated 1 × 10^7^ and 10^8^ CFU/g in the diets [15,17,18]. The final concentration of bacteria was determined using the serial dilution method in each agar broth [19].

### 2.2. Experimental Diets

Feed formulation and proximate composition of the basal diet is shown in Table 1. A basal control diet without supplementation of probiotics (CON), and five other diets including supplementation of *Bacillus subtilis* at 1 × 10^7^ CFU/g diet (BS_7_), *B. subtilis* (BS_8_), *Pediococcus pentosaceus* (PP_8_) and *Lactococcus lactis* (LL_8_) at 1 × 10^8^ CFU/g diet, and oxytetracycline (OTC) at 4 g/kg diet were used in this study. Fishmeal, soybean meal, wheat gluten meal, and squid liver powder were used as the protein sources. Fish oil served as the lipid source, wheat flour and corn starch as the carbohydrate sources. The preparation and storage of experimental diets were conducted following Bai & Kim [20]. Briefly, all the dry ingredients were weighted and mixed in a mixing machine, followed by the addition of fish oil and water, until a dough was formed. Experimental diets were pelleted using a laboratory pelleting machine with a 1 to 2-mm diameter module (Baokyong Commercial Co., Busan, Republic of Korea). The pellets were air-dried for 72 h and then stored at −20 °C in the refrigerator until use.

### 2.3. Experimental Shrimp and Feeding Trial

The feeding trial was carried out at Pukyong National University and Feeds and Foods Nutrition Research Center (FFNRC) in Busan, Republic of Korea. Juvenile whiteleg shrimp were purchased from Palddak shrimp farm (Goseong, Republic of Korea) and were transported to the laboratory. Shrimp were acclimatized to the FFNRC for two weeks and fed a basal diet. Prior to the feeding trial, shrimp were examined for external abnormalities and withheld for 24 h. At the beginning of the experiment, shrimp with the initial body weight of 1.41 ± 0.05 g (mean ± SD) were distributed into 18 (45 L) capacity rectangular tanks (20 shrimp/tank) at a constant flow (1.8 L/min) of filtered seawater. Each tank was randomly distributed into one of the three replicates of the eight dietary treatments. Shrimp were fed four times a day (09:00, 13:00, 17:00 and 21:00 h) at 5%–6 % of wet body weight/day for 8 weeks. Supplemental aeration was provided to stabilize the dissolved oxygen. The temperature of the aquarium was maintained at 27.0 ± 1.0 °C and pH remained at 7.68 ± 0.05. The condition of the tanks was maintained by siphoning off uneaten feeds 2 h after feeding and the walls and bottom of the tanks were scrubbed once a week.

### 2.4. Sample Collection and Analysis

At the end of the feeding trial, all of the shrimp were withheld for 24 h. The count and weight of total shrimp in each tank was measured to calculate final body weight, weight gain, feed efficiency, specific growth rate, protein efficiency ratio, and survival. Four shrimp from each treatment group were sacrificed to collect the blood samples. Serum samples were separated by centrifugation at 8000× *g* for 15 min and stored at −70 °C for the analysis of non-specific immune responses, including superoxide dismutase, myeloperoxidase, and lysozyme activity, as well as biochemical parameters such as aspartate aminotransferase activity, aminotransferase activity, total protein, and glucose. In addition, the anterior intestine of shrimp samples were collected for histological sections and digestive enzyme activity measurements.

The proximate composition of experimental diets and whole-body samples were analyzed following the standard methods of AOAC [21]. Moisture content was measured after oven-drying the samples at 105 °C to constant weight, while crude ash was estimated after incineration at 550 °C for 3 h. Crude protein was measured using the Kjeldahl method (N × 6.25) after acid digestion, and crude lipid was determined by Soxhlet extraction using the soxhlet system 1046 (Tacator AB, Hoganas, Sweden) after freeze-drying the samples for 20 h.

### 2.5. Non-Specific Immune Responses Analysis

Lysozyme activity was measured by supplementing 0.1 mL serum sample to *Micrococcus lysodeikticus* (0.2 mg/mL, Sigma) in a 0.02 M sodium phosphate buffer (pH 5.52). The reactions were performed at room temperature (20 °C) and the absorbance of the sample at a wavelength of 450 nm was measured between 0.5 min and 4.5 min with a spectrophotometer. The sample unit was defined as the amount of enzyme yielding a decrease in absorbance of 0.001/min. Superoxide dismutase activity was obtained by the superoxide radical dependent reaction inhibition rate of enzyme with Water Soluble Tetrazolium dye substrate and xanthine oxidase with the Superoxide dismutase Assay Kit (Sigma-Aldrich, 19160, St. Lousis, MO, USA), according to the manufacturer’s instructions. Each endpoint assay was estimated at 450 nm absorbance after incubating for 37 °C at 20 min. The percentage of inhibition was normalized by mg protein and expressed as Superoxide dismutase unit/mg. Additionally, myeloperoxidase activity was determined as described by Quade & Roth [22]. Briefly, 20 µL of serum was diluted with Hanks balanced salt solution (HBSS) without Ca^2+^ or Mg^2+^ (Sigma-Aldrich, USA) in separated 96 well plates to which 35 µL of 3, 3′, 5, 5′ tetramethylbenzidine hydrochloride (TMB; 20 mM; Sigma-Aldrich) and H_2_O_2_ (5 mM) were added afterwards. The color change reaction after 2 min was completed by adding 35 µL of 4 M sulphuric acid. The optical density was measured using a spectrophotometer at 450 nm.

### 2.6. Real-Time PCR

Five shrimp per experimental diet were used for sample analysis after anesthesia. Total RNA was extracted from mid intestine (50 mg) of shrimp using RiboEx™ (GeneALL, Seoul, Rep. Korea) following the standard procedures (Riboclear plus, GeneAll, South Korea). RNA concentration (ng/μL) and purity (OD 260:280) was determined with a nanodrop (Thremo Fisher Scientific, Waltham, MA, USA) and the 260/280 ratio was >1.8. The cDNA was synthesized from 1 μg of RNA using to the manufacturers’ instructions of cDNA synthesis Kit (Takara, Japan). RNA isolation and preparation of cDNA by 1 μg of RNA was performed following the manufacturer’s instructions (GeneAll, Korea). Then, the primer and target genes were prepared by the Bionics company (Seoul, Rep. Korea) (Table 2). Relative RNA level of the target genes was evaluated and calculated using endogenous β-actin RNA level.

### 2.7. Intestinal Histology

The mid-intestines of the shrimp (*n* = 3) were sampled from each experimental tank and were preserved in 10 % buffered formaldehyde for 24 h, then dehydrated in a graded ethanol series and embedded in paraffin. Tissue blocks were sectioned (5 μm) and stained with hematoxylin and eosin (H&E). The evaluation of villi height and muscular thickness was measured using a light microscope (AX70 Olympus, Tokyo, Japan) equipped with a scientific digital camera for microscopy (DIXI Optics, Daejeon, Rep.of Korea). The image was analyzed using the Image J 1.32j software (National Institute of Health, Bathesda, MD, USA). 

### 2.8. Challenge Test

*Vibrio parahaemolyticus* is a pathogen that commonly occurs in shrimp environments. The pathogenic bacterium, *V. parahaemolyticus* KCCM 11965, was obtained from the Department of Biotechnology, Pukyong National University, Busan, Republic of Korea. At first, bacteria was grown in 10 mL of brain heart infusion (BHI; Becton, Dickinson and Company, MD, USA) broth and incubated at 37 °C for 24 h with a shaking incubator. Growth of *V. parahaemolyticus* was observed by optical density of 600 (OD_600_ nm) using a spectrophotometer (Mecasys, Optizen, Republic of Korea), harvested by centrifugation and washed two times with 0.1 M PBS for further use. At the end of the experiment, eight shrimp from each tank were randomly collected and distributed based on their previous dietary treatment groups in 12 L tanks. Shrimp were injected intraperitoneally with 0.1 mL per shrimp of *V. parahaemolyticus* KCCM 11965 at 2 × 10^7^ CFU/mL (2 × LD_50_). Shrimp mortality was recorded daily up to 7 days and water temperature was maintained at 27 ± 1.0 °C (mean ± SD). 

### 2.9. Digestive Enzyme Activities

The enzyme activities of trypsin, lipase, and amylase were analyzed, following the manufacturer’s instructions, with enzyme assay kits (Biovision, Milpitas, CA, USA) and a spectrophotometer with the linear range. The pre-treatment of each specific enzyme assay kit was carried out with substrate and assay buffer. Trypsin activity was prepared with a mixed solution and measured by spectrophotometer at a wavelength of 405 nm for 40 min. Lipase activity was measured with a spectrophotometer at a wavelength of 412 nm for 20 min after mixing lipase substrate and assay buffer. Amylase activity was reacted with assay buffer and substrate mix, and was measured by absorbance of shrimp samples at a wavelength of 402 nm for 40 min. Specific enzyme activities were defined as the amount of enzyme that catalyzed the conversion of 1 μmol of substrate per minute per mg of protein (i.e., U mg soluble protein− 1) at the respective temperature. 

### 2.10. Statistical Analysis

The values from this study were statistically analyzed using one-way ANOVA (SAS Version 9.1, SAS Institute Inc., Cary, NC, USA) and in order to test the effects of dietary probiotic treatments. When a significant treatment effect was observed, an LSD post hoc test was used to compare means. Treatment effects were considered to be significant at *p* < 0.05.

## 3. Results

### 3.1. Growth Performance and Whole Body Proximate Composition

The growth performances and survival of juvenile whiteleg shrimp fed different probiotics diets are summarized in Table 3. Weight gain, specific growth rate, feed efficiency, and protein efficiency ratio of shrimp fed BS_8_ and LL_8_ diets were significantly higher than those of shrimp fed the CON diet (*p* < 0.05). However, there were no significant differences among shrimp fed BS_8_, PP_8_, LL_8_, BS_7_, and OTC diets (*p* > 0.05). Shrimp survival on the LL_8_ diet was significantly higher than for those shrimp on the CON and OTC diets (*p* < 0.05). However, there were no significant differences among shrimp fed BS_8_, PP_8_, LL_8_, and BS_7_ diets (*p* > 0.05). On the other hands, no significant differences were observed in terms of whole body protein, lipid, moisture, and ash content among all diets (*p* > 0.05; Table 4).

### 3.2. Non-Specific Immune Responses

The non-specific immune responses are shown in Table 5. Lysozyme activity in shrimp fed probiotics and OTC diets significantly improved compare to in shrimp on the CON diet (*p* < 0.05). Superoxide dismutase activity in shrimp fed BS_8_ and PP_8_ diets was significantly higher than that in shrimp fed the CON diet (*p* < 0.05). However, there were no significant differences among probiotics and OTC diets (*p* > 0.05). Meanwhile, myeloperoxidase did not show any significant differences among treatment diets (*p* > 0.05). 

### 3.3. Intestinal Histology

**Figure 1:**
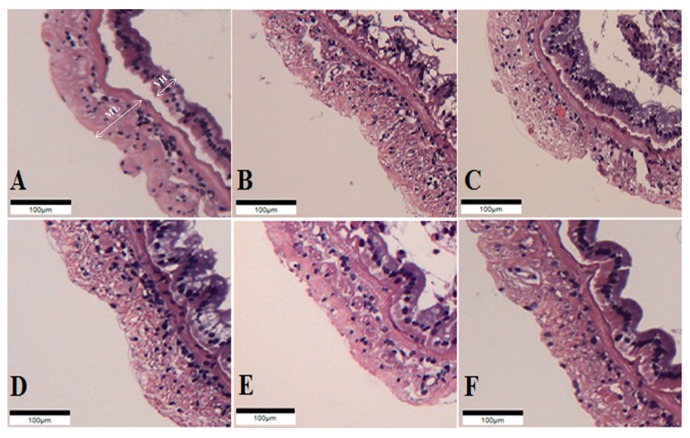
Histological sections of juvenile whiteleg shrimp intestine fed the experimental diets for 8 weeks. A-CON, basal diet; B-BS_7_, *Bacillus subtilis* at 1 × 10^7^ CFU/g; C-BS_8_, *Bacillus subtilis* at 1 × 10^8^ CFU/g; D-PP_8_, *Pediococcus pentosaceus* at 1 × 10^8^ CFU/g; E-LL_7_, *Lactococcus lactis* at 1 × 10^8^ CFU/g; F-OTC, oxytetracycline at 4 g/kg. (Scale bar = 100 µm; Original magnification × 4). ML = muscular layer thickness VH = villi height.

The intestinal histology of whiteleg shrimp fed experimental diets for 8 weeks was described (Figure 1, Table 6). The muscular layer thickness of shrimp fed the probiotic-supplemented diets was longer than those of shrimp fed the CON and OTC diets (*p* < 0.05). Correspondingly, the villi height of shrimp fed the PP_8_ and LL_8_ diets significantly improved compared to shrimp on the CON diet (*p* < 0.05). However, there were no significant differences among shrimp fed the BS_7_, BS_8_, PP_8_, LL_8_, and OTC diets (*p >* 0.05).

### 3.4. Haematological Analysis

As shown in Table 7, there were no significant differences among treatment groups in terms of aspartate aminotransferase activity, aminotransferase activity, glucose and total protein (*p >* 0.05). 

### 3.5. Immune-Related Gene Expressions

**Figure 2:**
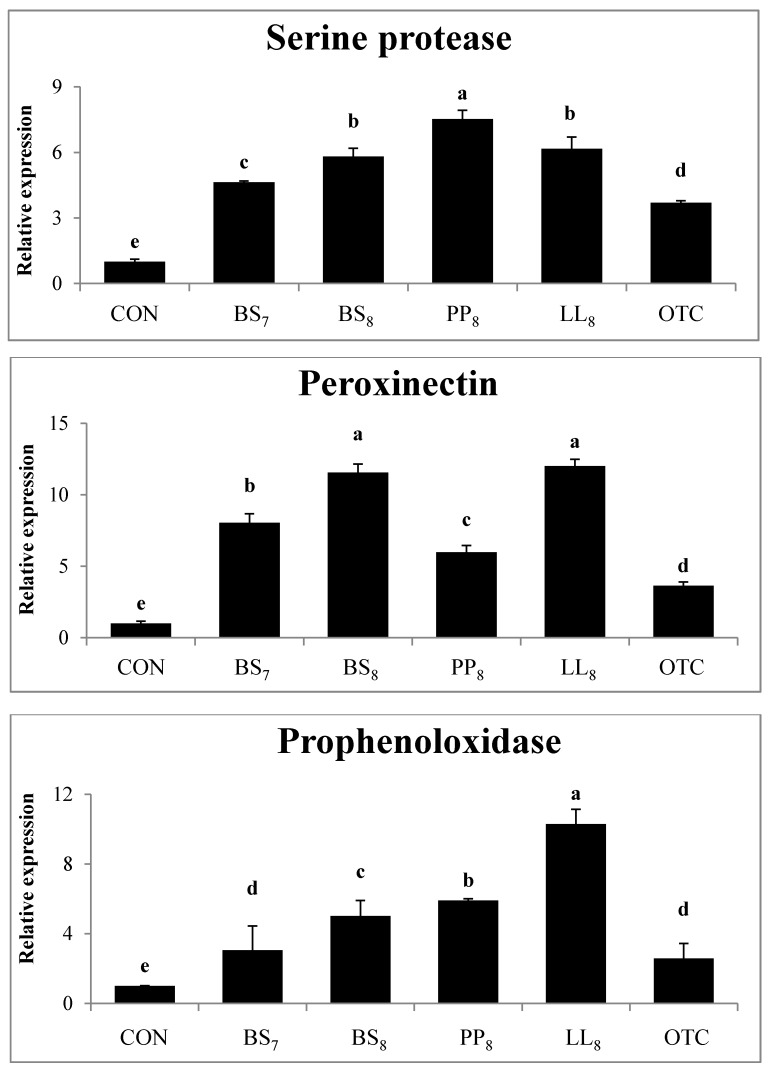
Intestinal gene expression levels of serine protease, peroxinectin and prophenoloxidase were evaluated in juvenile whiteleg shrimp fed the experimental diets for 8 weeks. Bars with range represent mean ± SD of triplicate samples, and diets refer to Figure 1.

The immunological gene expressions in the intestines of whiteleg shrimp fed experimental diets are demonstrated in Figure 2. The expression levels of serine protease from shrimp fed probiotic diets were significantly higher than those of shrimp fed the CON and OTC diets (*p* < 0.05). Also, the peroxinectin expression in shrimp fed probiotic diets was higher compared to in shrimp on the CON and OTC diets (*p* < 0.05). Meanwhile, prophenoloxidase expression in shrimp fed the BS_8_, PP_8_, and LL_8_ diets were significantly improved compare to those on the CON, BS_7_, and OTC diets. However, there were no significant differences between BS_7_ and OTC diets (*p* > 0.05).

### 3.6. Challenge Test

**Figure 3:**
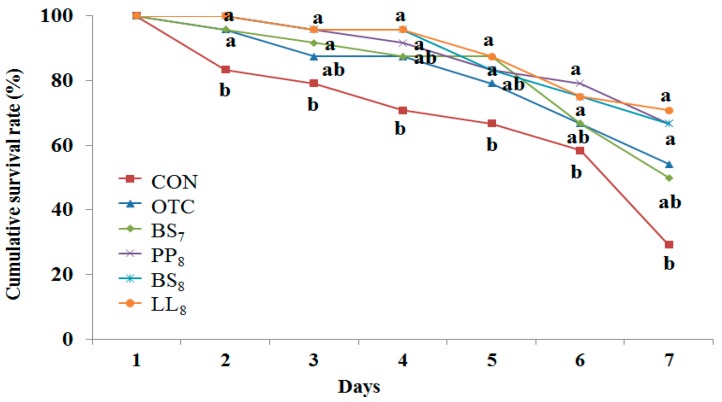
Cumulative survival rate of juvenile whiteleg shrimp fed the experimental diets with different probiotics for 8 weeks and experimentally challenged with *V. parahaemolyticus* for 7 days. Each value represents mean of triplicate groups. Significant differences among means are indicated by different superscripts (*p* < 0.05), and diets refer to Figure 1.

The cumulative survival rate of juvenile whiteleg shrimp challenged with *Vibrio parahaemolyticus* for 7 days is presented Figure 3. During the challenge test, the first shrimp mortalities occurred on the first day post-injection. Statistical analysis at the end of 7 days of the *V. parahaemolyticus* challenge showed that shrimp fed the BS_8_, LL_8_, and PP_8_ diets had significantly higher cumulative survival rates than those of shrimp fed the CON diet (*p* < 0.05). However, there were no significant differences among probiotic and OTC diets (*p >* 0.05).

### 3.7. Digestive Enzyme Activities

**Figure 4:**
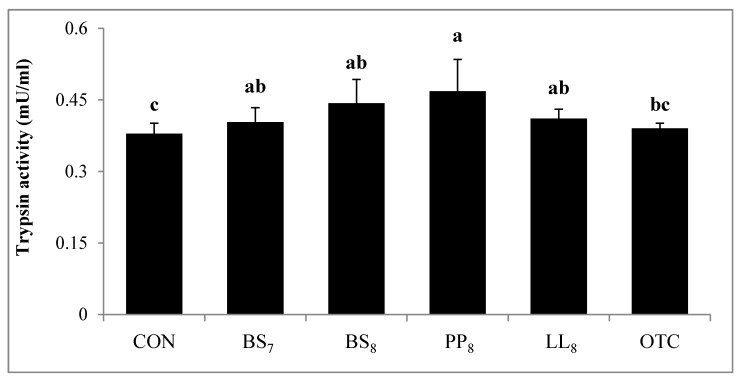

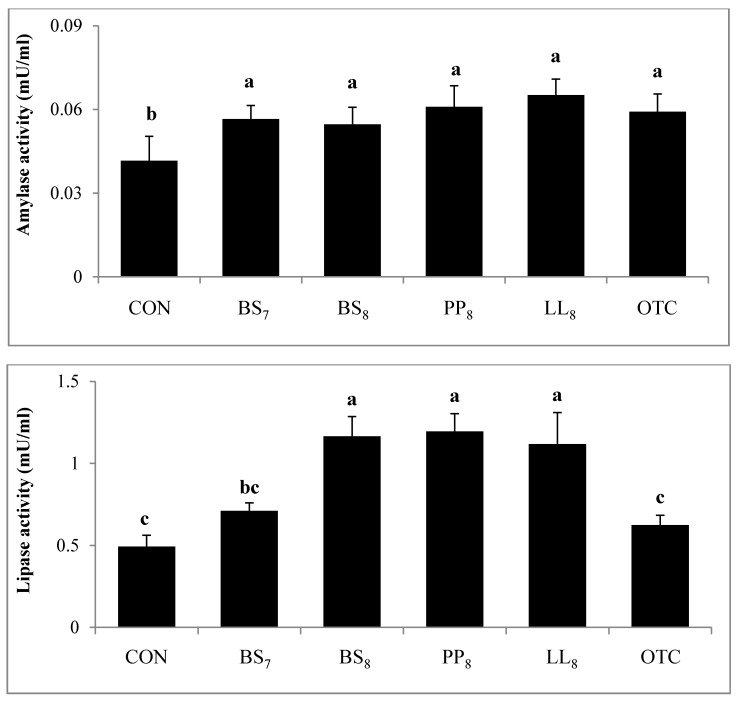
Specific enzyme activities of 1. Trypsin, 2. Amylase and 3. Lipase measured in the intestines of juvenile whiteleg shrimp fed the experimental diets with different probiotics for 8 weeks, and diets refer to Figure 1.

Digestive activity of juvenile whiteleg shrimp is shown in Figure 4. Trypsin activity in shrimp fed the BS_8_ and PP_8_ diets were significantly higher than for those on the CON diet (*p* < 0.05). However, there were no significant differences among shrimp on the BS_8_, PP_8_, LL_8_, BS_7_, and OTC diets (*p* > 0.05). Lipase activity of shrimp fed the BS_8_, PP_8_, and LL_8_ diets were significantly improved compared to those on the CON, BS_7_, and OTC diets (*p* < 0.05). Moreover, amylase activity in probiotic and OTC groups were significantly higher than the CON group (*p* > 0.05).

## 4. Discussion

The application of probiotics in shrimp diets have shown beneficial effects on the growth [23], immune response [24], and disease resistance [25]. Among the probiotics used in the present study, *B. subtilis* WB60, which was isolated from the intestine of juvenile Japanese eel *Anguilla japonica*, was reported as a potential probiotic, as well as an antibiotic replacement in the diet [18]. In this study, the effects of three different dietary isolated probiotic supplementations on growth, immune responses, histology, and gene expression in whiteleg shrimp were investigated. 

The results of our study indicated that dietary *B. subtilis* and *L. lactis* at 10^8^ CFU/g had a significant influence on the growth and feed utilization of whiteleg shrimp. Similar results were seen in previous studies on improved growth and feed utilization by probiotic supplementation such as *B. subtilis* [13], *L. lactis* [17] and *P. pentosaceus* [15], which were improved compared to the CON diet. The reasons behind the improved growth performance through probiotic supplementation could be explained through two hypotheses. The increased growth performance and feed-utilization of whiteleg shrimp fed probiotics may be due to enhanced: (1) gastrointestinal performance [26,27]; and (2) immune response [24,25]. 

The digestive enzyme activities of shrimp are important indicators for estimating the organism’s ability to metabolize given nutrients [28,29]. The results of this study indicated that BS_8_, PP_8_, and LL_8_ diets significantly improved trypsin, amylase, and lipase activity of the shrimp intestine compared to those on the CON diet (*p* < 0.05). A few studies conducted on shrimp with probiotic supplementation with *Bacillus* sp. [30,31], *L. lactis* [17], *Lactobacillus plantarum* [32], and *P. acidilactici* [33] demonstrated the response similar to our results. Furthermore, our results are similar to the observations of Javahery et al. [34], who demonstrated that the administration of probiotics leads to enhanced digestion and nutrient absorption in the shrimp intestine, which was stimulated by endogenous enzymes produced by probiotics. Also, probiotic supplementation can improve the digestion of protein, starch, and fat in whiteleg shrimp compared to the CON diet, as a result of the increased value of enzyme activity [15]. According to Zheng et al. [35], probiotic supplementation could improve the height and density of intestinal enterocytes. Moreover, the microvilli of the intestine extensively contributes to the absorptive ability of nutrients and expansion of surface area [36]. The intestinal histology has been determined to estimate the gut condition [37]. The muscular layer thickness of whiteleg shrimp fed probiotic diets was significantly higher in the mid-intestine than those of shrimp fed CON and OTC diets (*p < 0.05*). On the other hands, villi height was significantly higher in the mid-intestine of PP_8_ and LL_8_ diets compared to the CON diet (*p < 0.05*). However, there were no significant differences between supplemented-probiotic and OTC diets (*p > 0.05*). Our findings are in agreement with [35], who reported that enhanced villi height and muscular layer thickness have been associated with probiotic administration, which could improve its nutrient absorptive ability. As it was mentioned before, the improved growth performance, feed utilization, and survival of shrimp may be due to enhancing digestive enzyme activity and histological value induced by the probiotics. Meanwhile, the improved growth can be explained in view of enhanced immune response. The invertebrates rely on a non-specific immune system against pathogenic organisms, and it has been shown that it can be reacted by probiotics which generate transduction signaling molecules that have the ability to alert the immune responses by pathogenic bacteria [38]. Modulation and disease prevention of the immune system have been shown to be among the beneficial effects of probiotics for aquatic animals [31,39]. Various studies have revealed that probiotic supplementation can improve immune responses in the shrimp diet [25,40,41]. In the current study, the dietary probiotic groups improved in lysozyme activity compared to the CON diet (*p* < 0.05), whereas there were no significant differences with the OTC diet (*p* > 0.05). Moreover, Superoxide dismutase activity of shrimp fed BS_8_ and PP_8_ diets were significantly higher than those of shrimp fed the CON diet (*p* < 0.05), and this did not differ between the probiotic and OTC diets (*p* > 0.05). Previous studies have observed that the administration of probiotics that complement each other and occupy different niches within the gastrointestinal tract could result in improvement or prolongation of the desirable effects on the host immune system and health [42,43]. 

The prophenoloxidase, serine protease, and peroxinectin serve important roles in immune responses for crustaceans. The proposed mechanism of prophenoloxidase is that active phenoloxidase induces oxidation of phenols to quinones, and results in the production of melanin, which can hold and barricade infectious pathogens [44]. Consequently, this system leads to induced phagocytosis, cytotoxic reactant production, and anti-oxidant defense enzymes. In the current experiment, the BS_8_, PP_8_, and LL_8_ diets significantly improved prophenoloxidase, serine protease and peroxinectin production compared to the CON and OTC diets (*p* < 0.05). The immune gene expression of shrimp increased with probiotic supplementation, as shown in previous studies [12,25]. Further, Chiu et al. [25] reported that the expression of the prophenoloxidase system can elevate the molecular activity of cell adhesion [45], opsonin [46], degranulation [47], and peroxidase [48] of shrimp. These biological activities were achieved in the present study with the probiotic treatments and gene up-regulation was enhanced in treated shrimp compared to CON diet (*p* < 0.05). 

Disease resistance against *V. parahaemolyticus,* which generates the common disease of whiteleg shrimp [49], was significantly enhanced for 7 days by administration of the *B. subtilis*, WB*60*, *P. pentosaceus* and *L. lactis* at 1 × 10^8^ CFU/g diet. This result is in agreement with previous experiments affected by pathogens [12,24,41]. Generally, administration of probiotics in the shrimp diet was shown to decrease mortality rates compared to the CON diet [40,50,51]. Previous studies demonstrated that probiotic supplementation can be used for modulating fish health and disease resistance [52,53]. Indeed, probiotics can beneficially influence the disease resistance of fish to pathogen bacteria by producing antimicrobial substances and competing with pathogens for physical occupation of space [54]. As a result, the enhanced survival and cumulative survival rates could be due to probiotic supplementation.

Basically, the selection of endogenous or exogenous probiotics should be done after their evaluation to colonize, establish, and multiply in the fish gut. Endogenous probiotics are already accustomed to the environmental conditions of the host, while inappropriate exogenous probiotics can cause undesirable effects in the host [55]. In this study, endogenous *L. lactis* and *P. pentosaceus,* and exogenous *B. subtilis* WB60, were evaluated, and it was shown that the exogenous bacteria can also be effective in whiteleg shrimp. Further, the studies on dietary endogenous or exogenous *Bacillus* strains have demonstrated beneficial effects on growth, immune response, and disease resistance in shrimp [12,56,57]. 

In conclusion, the present study demonstrated that *L. lactis* at 1 × 10^8^ CFU/g could be an ideal probiotic in terms of growth performance, immune response, histology, immune-related gene expression, digestive enzyme activity, and disease resistance for whiteleg shrimp. Besides, potential probiotic *B. subtilis* WB60 and *P. pentosaceus* at 1 × 10^8^ CFU/g could also be beneficial probiotics and replace antibiotics in whiteleg shrimp. Further, the results of this study could suggest that these probiotics have the potential for biofloc application to improve growth and immune responses in shrimp farms.

## Figures and Tables

**Table 1:** Formulation and composition (% dry matter) of the basal diet for whiteleg shrimp.

Ingredients	%
Fishmeal, Chile ^1^	30.0
Soybean meal ^2^	25.0
Wheat flour ^1^	12.0
Wheat gluten meal ^1^	8.00
Corn starch ^1^	7.00
Squid liver powder ^1^	4.00
Fish oil ^3^	4.00
Calcium phosphate ^4^	2.50
Lecithin ^1^	2.00
Vitamin premix ^5^	2.00
Mineral premix ^6^	2.00
Cholesterol	0.50
Cellulose ^4^	1.00
**Proximate Composition**	
Moisture	9.60
Crude protein	42.8
Crude lipid	9.72
Crude ash	8.15

^1^. The Feed Co., LTD. Seoul, Rep. of Korea. ^2^. CJ cheiljedang Co., LTD. Seoul, Rep. of Korea. ^3^. Jeil Feed Co., LTD. Hamman, Rep. of Korea. ^4^. Sigma-Aldrich Korea, Yongin, Rep. of Korea. ^5^. Contains (as mg/kg in diets): Thiamine mononitrate, 15; Niacin, 150; dl-Calcium pantothenate, 150; Pyridoxine HCl, 15; Rivoflavin, 30; Biotin, 1.5; Folic acid, 5.4; Cobalamin, 0.06; Ascorbic acid, 300; Inositol, 150; Choline bitate, 3000; Retinyl acetate, 6; dl-α-Tocopherol acetate, 201; Menadion, 6. ^6^. Contains (as mg/kg in diets): Ca(IO)_3_, 0.0006; NaCl, 437.4; MgSO_4_·7H_2_O, 1379.8; NaSeO_3_, 0.00025; MnSO_4_, 0.016; ZnSO_4_·7H_2_O, 226.4; Fe-Citrate, 299; CuSO_4_, 0.00033; FeSO_4_, 0.0378; MgO, 0.00135.

**Table 2:** Primers used to quantify relative gene expression.

Primers	Sense	Sequences
Serine protease (AU_368151.1)	F	5′-CCGTCTTGGAGAATACGACTTGAG-3′
	R	5′-GCTACAGGTAGGCTGGATAACTTG-3′
Peroxinectin (AF188840.1)	F	5′-GTGAACGGTAGTCCTTTACCTAAT-3′
	R	5′-CGAGGTCCATAGAAAGCATCTC-3′
Prophenoloxidase (EU284136)	F	5′-CAAGCCCTTCGACTACCATATAC-3′
	R	5′-CTGACTGTTCACTTGAGTTCCC-3′
Actin (AF100986)	F	5′-TGGCAATGAGAGGTTCCG-3′
	R	5′-TGCTGTTGTAGGTGGTTTCG-3′

**Table 3:** Growth performance and feed utilization of juvenile whiteleg shrimp fed the experimental diets for 8 weeks^1^.

	Diets					
	CON	BS_7_	BS_8_	PP_8_	LL_8_	OTC
Initial body weight (g)	1.40 ± 0.05	1.42 ± 0.08	1.40 ± 0.09	1.42 ± 0.06	1.39 ± 0.03	1.42 ± 0.08
Final body weight (g)	6.42 ± 0.12 ^b^	6.67 ± 0.44 ^ab^	7.08 ± 0.51 ^a^	6.85 ± 0.14 ^ab^	7.15 ± 0.26 ^a^	6.71 ± 0.65 ^ab^
Weight gain (%) ^2^	359 ± 22.1 ^b^	373 ± 24.8 ^ab^	406 ± 28.7 ^a^	378 ± 16.2 ^ab^	417 ± 29.8 ^a^	373 ± 24.8 ^ab^
Feed efficiency (%) ^3^	83.7 ± 4.57 ^b^	87.9 ± 5.82 ^ab^	94.6 ± 6.88 ^a^	88.7 ± 3.20 ^ab^	97.1 ± 6.47 ^a^	87.2 ± 6.49 ^ab^
Specific growth ratio (%/day) ^4^	2.72 ± 0.09 ^b^	2.79 ± 0.10 ^ab^	2.89 ± 0.10 ^a^	2.79 ± 0.06 ^ab^	2.93 ± 0.10 ^a^	2.77 ± 0.10 ^ab^
Protein efficiency ratio ^5^	2.31 ± 0.08 ^b^	2.58 ± 0.12 ^ab^	2.51 ± 0.09 ^a^	2.57 ± 0.08 ^ab^	2.59 ± 0.09 ^a^	2.60 ± 0.06 ^ab^
Survival ^6^	73.3 ± 5.00 ^b^	76.7 ± 2.89 ^ab^	83.3 ± 5.00 ^ab^	76.7 ± 5.00 ^ab^	88.3 ± 2.89 ^a^	73.3 ± 5.77 ^b^

^1^ Data are means ± SD of triplicate groups of shrimp. Values in each row with different superscripts are significantly different (*p* < 0.05) Diets: CON = the basal diet, refer to Table 1; BS_7_ = *Bacillus subtilis* at 1 × 10^7^ CFU/g; BS_8_ = *Bacillus subtilis* at 1 × 10^8^ CFU/g; PP_8_ = *Pediococcus pentosaceus* at 1 × 10^8^ CFU/g; LL_8_ = *Lactococcus lactis* at 1 × 10^8^ CFU/g; OTC = oxytetracycline at 4 g/kg. ^2^ Weight gain (%) = [(final wt. - initial wt.) × 100]/initial wt. ^3^ Feed efficiency ratio (%) = (wet weight gain/dry feed intake) × 100. ^4^ Specific growth rate (%/day) = [(log_e_ final wt. - log_e_ initial wt.) × 100]/days. ^5^ Protein efficiency ratio = (wet weight gain/protein intake). ^6^ Survival (%) = [(total shrimp – dead shrimp) × 100]/total shrimp.

**Table 4:** Whole-body proximate composition (% dry matter) of juvenile whiteleg shrimp fed the different probiotics diets for 8 weeks ^1^.

	Diets					
	CON	BS_7_	BS_8_	PP_8_	LL_8_	OTC
Moisture	75.4 ± 1.08	76.0 ± 1.24	75.2 ± 1.10	75.8 ± 1.28	75.5 ± 1.32	76.2 ± 1.05
Protein	17.8 ± 0.45	18.4 ± 0.33	17.9 ± 0.29	18.2 ± 0.52	18.7 ± 0.44	18.6 ± 0.38
Lipid	2.25 ± 0.08	2.16 ± 0.10	2.19 ± 0.05	2.20 ± 0.07	2.21 ± 0.03	2.22 ± 0.05
Ash	3.54 ± 0.12	3.62 ± 0.10	3.60 ± 0.09	3.58 ± 0.12	3.62 ± 0.08	3.55 ± 0.10

^1^ Data are means ± SD of triplicate groups of shrimp. Values in each row with different superscripts are significantly different (*p* < 0.05).

**Table 5:** Non-specific immune responses of juvenile whiteleg shrimp fed the experimental diets for 8 weeks ^1^.

	Diets					
	CON	BS_7_	BS_8_	PP_8_	LL_8_	OTC
Lysozyme (U/mL)	0.20 ± 0.03 ^b^	0.32 ± 0.02 ^a^	0.32 ± 0.05 ^a^	0.34 ± 0.04 ^a^	0.34 ± 0.02 ^a^	0.30 ± 0.03 ^a^
Superoxide dismutase (% inhibition)	93.9 ± 2.15 ^b^	95.6 ± 1.38 ^ab^	98.6 ± 3.69 ^a^	98.7 ± 2.66 ^a^	95.8 ± 2.71 ^ab^	94.7 ± 2.28 ^ab^
Myeloperoxidase (OD at 450 nm)	3.41 ± 0.40	3.76 ± 0.38	3.81 ± 0.09	3.77 ± 0.21	3.74 ± 0.29	3.69 ± 0.33

^1^ Values are means ± SD of triplicate groups of shrimp. Values in each row with different superscripts are significantly different (*p* < 0.05). Diets refer to Table 3.

**Table 6:** Intestinal histology of juvenile whiteleg shrimp fed the different probiotics diets for 8 weeks ^1^.

	Diets					
	CON	BS_7_	BS_8_	PP_8_	LL_8_	OTC
Muscular layer thickness (μm)	134 ± 13.9 ^d^	225 ± 15.2 ^c^	365 ± 37.5 ^a^	320 ± 19.0 ^b^	331 ± 52.0 ^ab^	129 ± 25.7 ^d^
Villi height (μm)	87.6 ± 11.9 ^b^	107 ± 5.54 ^ab^	108 ± 19.4 ^ab^	112 ± 17.8 ^a^	121 ± 17.8 ^a^	99.2 ± 13.7 ^ab^

^1^ Data are means ± SD of triplicate groups of shrimp. Values in each row with different superscripts are significantly different (*p* < 0.05). Diets refer to Table 3.

**Table 7:** Haematological analysis of juvenile whiteleg shrimp fed the different probiotics diets for 8 weeks ^1^.

	Diets					
	CON	BS_7_	BS_8_	PP_8_	LL_8_	OTC
Aspartate aminotransferase activity (U L^−1^)	118 ± 2.08	123 ± 4.16	113 ± 10.6	119 ± 2.89	122 ± 8.39	122 ± 2.89
Aminotransferase activity (U L^−1^)	175 ± 12.6	171 ± 18.0	2.89 ± 0.10	2.79 ± 0.06	2.93 ± 0.10	2.77 ± 0.10
Total protein (g dL^−1^)	11.3 ± 0.58	11.0 ± 1.00	10.7 ± 0.58	11.3 ± 0.58	11.3 ± 0.58	11.7 ± 0.58
Glucose	74.7 ± 5.13	76.3 ± 4.04	71.3 ± 5.86	70.7 ± 5.13	69.3 ± 5.69	75.3 ± 3.06

^1^ Data are means ± SD of triplicate groups of shrimp. Diets refer to Table 3.
